# A_2A_ Adenosine Receptor: A Possible Therapeutic Target for Alzheimer’s Disease by Regulating NLRP3 Inflammasome Activity?

**DOI:** 10.3390/ijms23095056

**Published:** 2022-05-02

**Authors:** Stefania Merighi, Manuela Nigro, Alessia Travagli, Silvia Pasquini, Pier Andrea Borea, Katia Varani, Fabrizio Vincenzi, Stefania Gessi

**Affiliations:** 1 Department of Translational Medicine and for Romagna, University of Ferrara, 44121 Ferrara, Italy; mhs@unife.it (S.M.); manuela.nigro@edu.unife.it (M.N.); alessia.travagli@edu.unife.it (A.T.); vrk@unife.it (K.V.); fabrizio.vincenzi@unife.it (F.V.); 2 Department of Chemical, Pharmaceutical and Agricultural Sciences, University of Ferrara, 44121 Ferrara, Italy; psqslv@unife.it; 3 University of Ferrara, 44121 Ferrara, Italy; bpa@unife.it

**Keywords:** A_2A_ receptors, NLRP3 inflammasome, Alzheimer’s disease, memory loss, neurodegeneration

## Abstract

The A_2A_ adenosine receptor, a member of the P1 purinergic receptor family, plays a crucial role in the pathophysiology of different neurodegenerative illnesses, including Alzheimer’s disease (AD). It regulates both neurons and glial cells, thus modulating synaptic transmission and neuroinflammation. AD is a complex, progressive neurological condition that is the leading cause of dementia in the world’s old population (>65 years of age). Amyloid peptide-β extracellular accumulation and neurofibrillary tangles constitute the principal etiologic tracts, resulting in apoptosis, brain shrinkage, and neuroinflammation. Interestingly, a growing body of evidence suggests a role of NLRP3 inflammasome as a target to treat neurodegenerative diseases. It represents a tripartite multiprotein complex including NLRP3, ASC, and procaspase-1. Its activation requires two steps that lead with IL-1β and IL-18 release through caspase-1 activation. NLRP3 inhibition provides neuroprotection, and in recent years adenosine, through the A_2A_ receptor, has been reported to modulate NLRP3 functions to reduce organ damage. In this review, we describe the role of NLRP3 in AD pathogenesis, both alone and in connection to A_2A_ receptor regulation, in order to highlight a novel approach to address treatment of AD.

## 1. Introduction

Multiple neurodegenerative illnesses, such as Alzheimer’s disease (AD), Parkinson’s disease (PD), multiple sclerosis (MS), and amyotrophic lateral sclerosis (ALS), all include neuroinflammation as a critical factor in their genesis and progression [1,2,3]. However, in primary neurodegenerative disorders defined by the aggregation of misfolded proteins, such as AD and PD, it is unclear whether inflammation is the underlying cause of disease or a response to pathology. Indeed, the pathophysiological explanation for neurodegenerative illnesses is based on the notion that certain proteins assemble into fibrils or oligomers when their conformations change, causing neurotoxicity and ending in neurodegeneration and inflammation [4,5,6,7]. Neuroinflammation is a protective response in the brain to external and endogenous insults to the central nervous system (CNS). However, exaggerated inflammatory responses can impair CNS homeostasis. The nucleotide-binding oligomerization domain leucine-rich repeat and pyrin domain-containing protein 3 (NLRP3) inflammasome, one of the most well-studied inflammasomes, plays a crucial role in neurodegenerative diseases, recognizing a variety of inflammation-inducing stimuli, such as damage-associated molecular patterns (DAMPs) or pathogen-associated molecular patterns (PAMPs), leading to the production of proinflammatory cytokines IL-1 and IL-18 [8]. Interestingly, a crucial role for the endogenous nucleoside adenosine through its A_2A_ receptor subtype has been reported in neuroinflammation [9]. The inhibition of the A_2A_ adenosine receptor has been shown to be beneficial in animal models of Huntington’s and Alzheimer’s disease, epilepsy, and excitotoxic situations such as ischemia, and it is also a strategy used to treat PD [10,11,12,13]. Furthermore, recent evidence indicates its ability to also regulate the NLRP3 inflammasome [14,15]. These encouraging results prompted us in this review to focus the attention on the involvement of NLRP3 in AD pathology alone and in relation to A_2A_ receptor modulation.

## 2. Adenosine and A_2A_ Receptor Activation

Adenosine is a widely distributed nucleoside that is produced mostly from the hydrolysis of ATP and operating as an extracellular autacoid to control a variety of pathological activities. The action of cytosolic 5′-nucleotidase on AMP or the activity of S-adenosylhomocysteine (SAH) hydrolase on SAH results in intracellular adenosine synthesis. When cells are subjected to increased energy metabolism or stress, such as in diseased situations, an extracellular route takes precedence. In this situation, the activity of certain enzymes known as ectonucleoside triphosphate diphosphohydrolases (CD39s), which metabolize ATP into AMP, and ecto-5′-nucleotidase (CD73), which dephosphorylates AMP to create adenosine, is the dominant contributor of adenosine [9]. The extracellular nucleoside enters cells via concentrative and equilibrative nucleoside transporters, CNTs (SLC28 family) and ENTs (SLC29 family), respectively, once it is produced. The ENTs perform a critical function in regulating adenosine content. The sodium gradient drives CNT proteins to promote adenosine passage from extracellular to intracellular stores, whereas ENTs enable bidirectional movement by following the nucleoside-level gradient across the membrane. These transporters typically allow extracellular adenosine to move into the cell, but this flow is opposite under hypoxia [16].

The enzyme adenosine deaminase (ADA), which converts extracellular adenosine to inosine, regulates the quantity of extracellular adenosine. Adenosine can be also transformed to inosine by intracellular ADA or phosphorylated to AMP by intracellular adenosine kinase (ADK). ADA possesses a lower affinity for the nucleoside in comparison to ADK, with the consequence that AMP synthesis is prevalent under normal settings, while in stressed circumstances, it is mostly metabolized to inosine [17]. The activation of four transmembrane receptors, known as A_1_, A_2A_, A_2B_, and A_3_ adenosine receptors (ARs), is responsible for adenosine responses [18]. In the field of AD, the most promising target inside the adenosine-receptor family is the A_2A_ subtype. It is coupled to Gs and Golf proteins to stimulate adenylyl cyclase activity through the body and in the striatum, respectively [19,20]. It also regulates the activation of important transduction pathways, such as p38, ERK1/2, JNK1/2 mitogen-activated protein kinases (MAPK) and Akt, possibly impacting a variety of pathophysiological processes [21,22].

The A_2A_ receptor is the most advanced adenosine subtype in terms of both knowledge of effects produced in neurodegenerative diseases as well as availability of selective receptor antagonists already entered in clinical studies. Indeed, the caffeine derivative istradefylline, the first commercially available selective A_2A_ receptor antagonist, is currently available for PD [23,24]. Caffeine, found in coffee, the world’s most consumed beverage, possesses a high affinity for the A_2A_ receptor subtype and can also help with cognition by lowering tau hyperphosphorylation in the hippocampus, reducing neuroinflammation, and recovering memory loss [23,25,26,27]. Importantly, a Phase 3 multicentre, randomized, double-blind, placebo-controlled clinical trial is underway to assess the effect of 30-week caffeine intake on cognition in individuals with mild-to-severe AD (CAFfeine on Cognition in Alzheimer’s Disease, CAFCA, NCT04570085). The findings of human studies will assist to define the role of caffeine in the management of this ailment. 

## 3. NLRP3 Inflammasome

**Figure 1:**
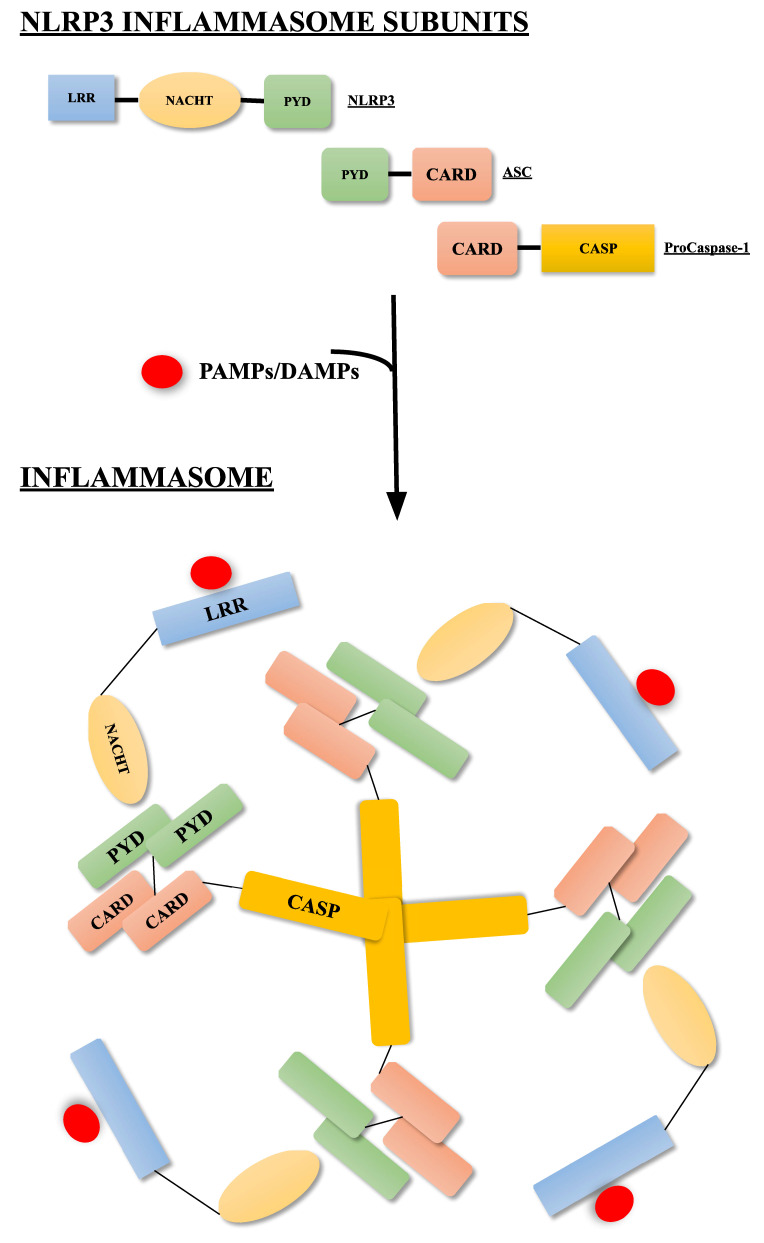
NRLP3 inflammasome subunits and their subsequent oligomerization after binding with PAMPs or DAMPs, as pathogens or intracellular danger signals. The NLRP3 inflammasome is a multiprotein complex including NLRP3, ASC and pro-caspase 1 domains. Following interaction with PAMPs and DAMPs, it undergoes oligomerization, through the binding to ASC and then caspase-1 precursors. Adaptor protein apoptosis-associated speck-like protein (ASC); caspase-activation recruitment domain (CARD); amino-terminal pyrin domain (PYD), nucleotide-binding domain (NACHT); carboxy-terminal leucine-rich repeat (LRR); caspase 1 (CASP); damage-associated molecular patterns (DAMPs); pathogen-associated molecular patterns (PAMPs).

The NLRP3 inflammasome is a cytosolic multiprotein complex constituted of NLRP3 protein, adaptor protein apoptosis-associated speck-like protein (ASC) containing a caspase activation and recruitment domain (CARD), and the precursor of caspase-1 (pro-caspase 1). Specifically, NLRP3 is a tripartite complex consisting of an amino-terminal pyrin domain (PYD) domain, a nucleotide-binding domain (NACHT, including NAIP, CIITA, HET-E and TP1 proteins) with ATPase activity, and a carboxy-terminal leucine-rich repeat (LRR) domain, mediating recognition stimulation. Under normal physiological conditions, the NACHT domain of NLRP3 binds to LRRs, resulting in autoinhibition [28,29,30]. In the presence of PAMPs or DAMPs it functions as a damage sensor to recognize pathogens and intracellular danger signals, undergoes oligomerization through binding to the adaptor protein ASC and then recruiting caspase-1 precursors [31,32]. Indeed, ASC is an adaptor molecule present in the nucleus containing PYD and CARD domains, that during stress conditions translocates into the cytoplasm, bridging NLRP3 and procaspase-1, thus activating the NLRP3 inflammasome aggregates. NLRP3 and ASC interact by PYD domains, while ASC and procaspase-1 by CARD domains (Figure 1) [30]. Finally, caspase-1 (IL-1β converting enzyme) is the effector protein of the NLRP3 inflammasome, converted from the precursor procaspase 1 to produce the active form. Caspase-1 then activates the precursor of pro-IL-1β and pro-IL-18 to give mature IL-1β and IL-18 inflammatory cytokines [31]. IL-1β increases fever, vasodilation, and adhesion-molecule expression, thus allowing migration of immune cells into injured tissues. In addition, it hampers microglial phagocytosis [33], provokes tau hyperphosphorylation [34], and modulates synaptic plasticity, thus impairing learning and memory functions [35,36]. In contrast, IL-18 regulates IFNγ production and Th17 cell inhibition, while it promotes Treg cell activity [37,38]. Interestingly, IL-18 increases Aβ production by human neuron-like cells, revealing its crucial role in AD development [39]. Interestingly, a gene polymorphism affecting the promoter region of human IL-18 is associated with the risk of AD [30]. In addition to cytokine modulation, caspase-1 may trigger an inflammatory type of programmed cell death named pyroptosis by activation of gasdermin D (GSDMD) [40,41,42,43]. During this process, plasma membrane pore formation is due to the interaction of the N-terminal fragment of GSDMD with phosphoinositides. This alters osmotic membrane potential, causing cellular swelling, cell lysis, and release of mature IL-1β and IL-18 [44,45]. NLRP3 is a crucial monitor of cellular injury, alerting towards alteration in cellular homeostasis. Its activation occurs through canonical and noncanonical modes of activation [30,42,46]. The first involves two-steps, named priming and activation. The “priming” signal results in the activation of the Toll-like receptor 4 (TLR-4)-induced NF-kB transcription pathway, leading to the transcription of NLRP3 and pro-IL-1β and pro-IL-18. The activation step is promoted by different stimuli, including viral RNA, ATP, pore-generating toxins, K^+^ efflux, elevated mitochondrial reactive oxygen species (ROS) generation, β-amyloid (Aβ), and α-synuclein, leading to the oligomerization and activation of the NLRP3 inflammasome with activated caspase-1 formation. The “noncanonical” NLRP3 activation pathway involves LPS-induced activation of TLR4 to induce caspase-11 transcription in mice (or its human orthologs caspase-4 and caspase-5). Alternatively, Gram-negative bacteria release outer membrane vesicles, which function as a vehicle for delivering LPS into the cytosol. The CARD domain of caspase-11 recognizes LPS in the cytosol directly, causing it to oligomerize. The active caspase-11 subsequently cleaves the pore-forming protein GSDMD between the N-terminal and C-terminal domains. The N-terminal domain of GSDMD generates pores in the plasma membrane, which facilitates potassium efflux, pyroptosis, and subsequent NLRP3 inflammasome activation. Thus, caspase-11 does not directly cleave interleukins but through induction of pyroptosis, and consequent potassium efflux triggers NLRP3 inflammasome and caspase-1 activation, which in turn is responsible for IL-1β secretion [45,47]. This picture demonstrates the interplay between the canonical and noncanonical inflammasome modes of activation.

## 4. Inflammasome and AD

AD is the most prevalent age-related neurodegenerative condition, characterized by aberrant protein aggregation of Aβ plaques and neurofibrillary tangles made up of hyperphosphorylated tau [48,49]. These protein clumps are first located in the neocortex, then move to the entorhinal cortex and hippocampus with time [50]. The activation of microglia and astrocytes, which increases the production of inflammatory mediators to help with cellular repair as well as stimulate the phagocytosis of undesirable debris, correlates with the formation of aggregated protein, as it does in many other neurodegenerative conditions. However, prolonged activation of microglia and increased production of inflammatory markers, notably IL-1β, have been reported in microglial cells near Aβ plaques, in AD patients, and in animal models of illness [51,52]. Furthermore, higher levels of IL-1β and IL-18 have been observed in cerebrospinal fluid of AD patients [53]. It is known that both aggregated protein deposition and immune-cell over-stimulation can affect neuron structure and function, causing episodic memory loss and cognitive impairment, which are hallmarks of AD [48,54,55,56,57].

The NLRP3 inflammasome has been linked to the innate immune response in AD [58,59]. The data reported in this review offer a proof of concept for considering NLRP3 inflammasome modulation as a treatment option to slow the course of this illness. Increased expression of NLRP3 in AD mice or a human microglia cell line or in the brain of AD patients has been observed [60,61,62]. Specifically, NLRP3 inflammasome activation occurs following phagocytosis of fibrillar Aβ by murine-cultured microglial cells, causing lysosomal destruction, cathepsin B release, and caspase-1 activation, as well as IL-1β and TNF-α secretion, and nitric oxide production [60].

Indeed, acute microglial recruitment in AD brain slows disease development by encouraging Aβ clearance before senile plaque formation. Nevertheless, the capacity of microglia to remove Aβ has been found to decline with age and advancement of AD pathology, as seen by persistent Aβ accumulation despite growing microglial numbers. Accordingly, microglia from old—but not young—AD transgenic mice have fewer Aβ-binding scavenger receptors and Aβ-degrading enzymes, indicating that these cells become more proinflammatory and miss their Aβ-clearing abilities, culminating in decreased Aβ uptake and degradation, as well as enhanced Aβ accumulation [63]. Therefore, this dualistic microglial function should be addressed when anti-inflammatory treatment for AD is considered.

Recently, it has been observed that lowering the expression of microglia CX3CR1, a receptor involved in its recruitment towards inflammatory sites, or partially inhibiting its activity in the brain may be a therapeutic strategy to increase neuronal Aβ clearance, reduce Aβ levels, and delay progression of dementia [64]. In a transgenic mouse model of AD, NLRP3 or caspase-1 knockdown prevented spatial-memory impairments and decreased brain concentrations of caspase-1 and IL-1β, while increasing microglial phagocytosis able to remove Aβ, suggesting a role of NLRP3 inflammasome in the pathogenesis of AD. NLRP3 activation negatively impacts the phagocytic function of microglia in AD. Importantly, poor clearance of Aβ may be the underlying cause of sporadic AD, which accounts for the vast majority of human AD cases. In addition, decreased inflammasome activity promotes the switch of microglia cells towards an anti-inflammatory M2 phenotype, reducing Aβ deposition [61]. Interestingly, it has been reported that 3 months of peripheral immune stimulation of aged and AD—but not young—adult mice can provoke permanent impairment of learning and memory functions, due to a reduction in neuronal complexity and spine density of hippocampal neurons [65]. In addition, in APP/PS1 animals it was found that ASC specks secreted by microglia, following pyroptosis, promptly interacted with Aβ, boosting Aβ-oligomer production and the development of AD disease, while its deficiency was able to prevent it [66]. These results show that Aβ may engage the NLRP3 inflammasome in microglia, promoting the continuous production of proinflammatory cytokines and ASC specks, thus supporting the progression of AD pathology. Accordingly, a small-molecule inhibitor of NLRP3, named MCC950, ameliorated cognitive function, decreased Aβ deposition, and raised Aβ clearance in a mouse model of AD [67].

Moreover, IL-1β release, NLRP3 activation, and consequent neuroinflammation were reduced when AD transgenic mice were treated with mefenamic acid, which belongs to the nonsteroidal anti-inflammatory class of drugs [68]. In addition, episodic and spatial memory deficiency, as well as neuroinflammation, were improved through caspase-1 activity inhibition in a mouse model of AD [69]. In line with these findings, a higher quantity of active caspase-1 was observed in brain lysates from AD and moderate cognitive-impairment (MCI) patients compared to healthy subjects, indicating chronic inflammasome activity in these pathologies [61,70]. As for human studies, it has been found that increased gene expression of NLRP3, as well as ASC, caspase-1, and the cytokines IL-1β and IL-18, have been detected in cultured monocytes from AD patients, but not from healthy subjects, stimulated with LPS and Aβ peptide, suggesting that the peripheral NLRP3-mediated immune response is amplified in illness [36]. It has been reported that peripheral monocytes/macrophages are able to enter the brain, representing an important factor in the neuroinflammation associated with AD [71,72]. Indeed, animal models of AD confirm the presence of bone-marrow-derived macrophages near Aβ plaques [73]. Nevertheless, further longitudinal studies are necessary to determine if their effect includes an upsurge in inflammation or the removal of Aβ deposits from the brain in an attempt to lower Aβ-plaque buildup. However, even though the NLRP3 inflammasome drives AD pathology in transgenic mice, surprisingly, IL-18 prevented the development of lethal seizures by increasing neuronal transmission [74]. Therefore, while blocking the NLRP3 inflammasome could be one way to slow AD development, it is important to keep in mind that inhibiting particular cytokines in the brain could have unforeseen negative repercussions.

An elevated level of neuronal tau hyperphosphorylation is another result of NLRP3 activation [75,76]. Previously, a role for microglia in tau seeding and spreading was suggested [77,78]; in particular, it has been shown that in AD, tau disease is preceded by the production of Aβ plaques, with NLRP3 activation occurring before tau pathology in Tau22 mice [76]. Thus, NLRP3-dependent tau hyperphosphorylation occurs in a way involving IL-1β. Conversely, tau molecules, by activating NLRP3, have a significant impact on microglia. Because nonfibrillar tau may be actively secreted by neurons [79,80], it may play a role in persistent microglial activation, typically occurring in tauopathies. NLRP3 or tau binding to inhibitors of microglial activation might possibly disrupt this mechanism. Interestingly, it has been reported that miRNAs, small single-strand RNAs that silence messenger RNA translation, regulate NLRP3 inflammasome activation, thus decreasing transcript levels and changing protein expression [81]. A peculiar pattern of miRNA expression has been measured in AD brains [82,83,84,85,86]. Specifically, miR-7-5p, miR-22-3p, miR-30e, and miR-223-3p were found to bind the 3′UTR region of NLRP3 mRNA hampering protein translation and inhibiting the inflammasome protein complex formation [87,88]. Remarkably, in a pilot study involving 20 AD patients, PBMC expression of miR-7-5p and miR-223-3p was higher in tandem with increased activation of the NLRP3 inflammasome, suggesting that these miRNAs may be overexpressed in AD in a vain effort to suppress NLRP3 signaling, possibly implying that the brake of these molecules on NLRP3 is compromised in AD [89].

## 5. Adenosine A_2A_ Receptors and the Inflammasome in AD

**Figure 2:**
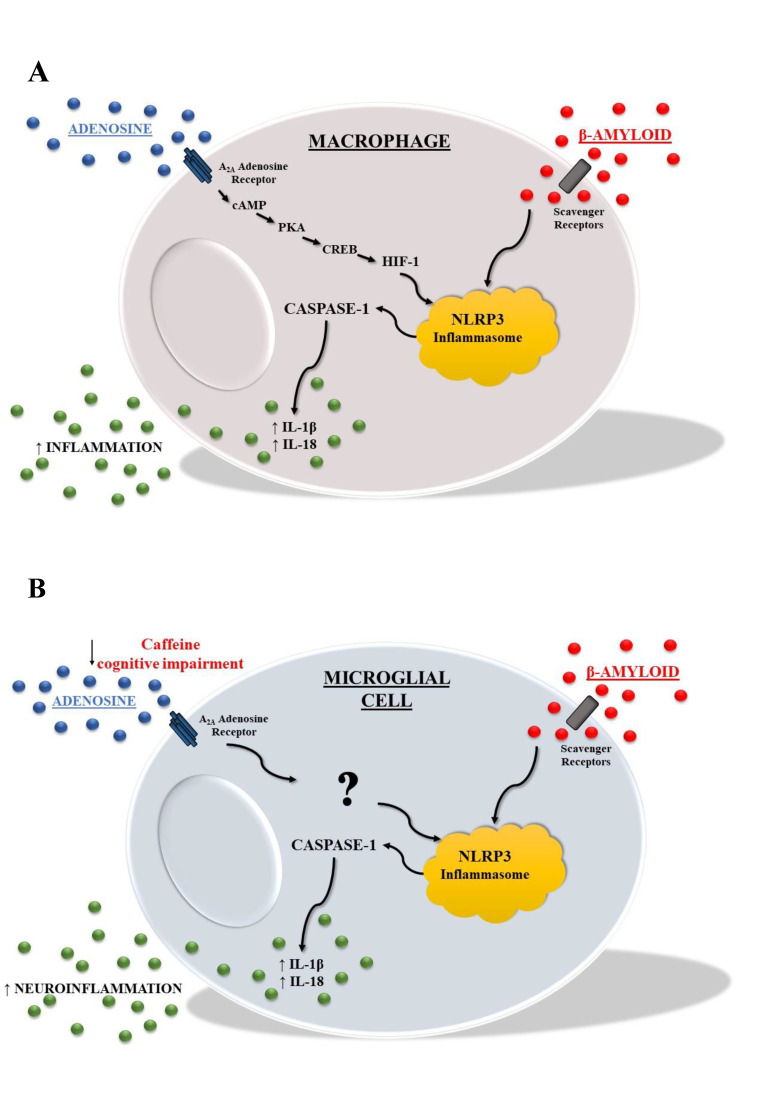
The picture represents the effect of A2A receptor activation on NLRP3 inflammasome function in macrophage (**A**) and microglia (**B**). (**A**) In macrophages, adenosine, increased during tissue injury, activates A2A receptor that through cAMP/PKA/CREB/HIF-1α signaling results in upregulation of NLRP3, Caspase-1, and IL1β/IL-18 production. (**B**) In microglia, adenosine, through A2A receptor, affects NLRP3 inflammasome and caffeine, and by blocking this signal may improve cognition. β-amyloid may engage the NLRP3 inflammasome, promoting production of proinflammatory cytokines.

While adenosine effects on neuroinflammation through A_2A_ receptor activation have been widely investigated, little is known about adenosine modulation of NLRP3 inflammasome machinery. A pioneering work by Ouyang et al. demonstrated that adenosine through the A_2A_ receptor is a crucial modulator of inflammasome activation in murine macrophages [90]. Specifically, adenosine controls inflammasome activity initiated by a variety of PAMPs and DAMPs, and A_2A_ receptor activation triggers a cAMP/PKA/CREB/HIF-1 signaling pathway, which leads the upregulation of pro-IL-1 and NLRP3, as well as increased caspase-1 activation (Figure 2A). Importantly, physiological levels of adenosine are required for optimal IL-1 secretion, and adenosine controls subsequent IL-1 production without the necessity for any initiating signal other than those mentioned above. This shows that these cells are not only tolerant or unresponsive to additional stimuli, but that they are in a postactivation state where they have shifted from a DAMP-driven to an adenosine-cAMP-driven phenotype [90]. These results have also been tested in “in vivo” using two models of liver injury, both of which demonstrated that A_2A_ receptor stimulation increases inflammasome activation in acute and chronic damage. This was an important demonstration of the magnitude of the adenosine signal in vivo. Homozygous A_2A_ receptor defective mice show more organ damage in different experimental models. This apparent paradox can be explained if adenosine increases macrophage-dependent inflammatory processes while also protecting parenchymal cells, both as part of an integrated response to tissue damage caused by pathogens and sterile assaults. Accordingly, in peripheral THP-1 macrophages it has been observed that caffeine reduced LPS-ATP-stimulated IL-1β and IL-18 secretion by decreasing NLRP3 expression and activation, ASC speck formation, and caspase 1 maturation. The signaling pathway activated by caffeine involved MAPK-dependent NF-kB inhibition by means of A_2A_ receptor antagonism [15]. In contrast, in a different experimental model using macrophages isolated from buffy coats of healthy donors and differentiated with M-CSF, it was reported that caffeine increased A_2A_ receptor expression and potentiated NLRP3-induced IL-1β secretion as well as caspase 1 activity, thus acting as a proinflammatory agent [91]. It has been suggested that the different cellular models and experimental conditions used for this research may account for the discrepancies reported. Indeed, the same paper reports that the comparative analysis of caffeine effects in macrophages differentiated with macrophage colony-stimulating factor (MM) or granulocyte-macrophage colony-stimulating factor (GM), leading to the development of inflammation-resolving (MM) or inflammation-promoting (GM) macrophages, respectively. Although caffeine reduced TNF-α secretion in both and had no effect on proinflammatory cytokine secretion in GM, it greatly increased the production of IL-6, IL-8, and IL-1β in MM, suggesting that the effects are strictly dependent by the specific populations under investigation [91]. The effects of caffeine have also been investigated in a model of hypoxic-ischemic white matter damage in neonatal rats where it inhibited, via A_2A_ receptor blockade, NLRP3 inflammasome activation and stimulated M2 polarization, thus preventing cerebral damage (Figure 2) [92]. Several lines of clinical research back up the idea that inflammation plays a role in brain damage and a decrease in neuroinflammation is considered a successful approach for the therapeutic treatment in premature infants [93,94]. Recently, the role of A_2A_ receptor stimulation has been deeply investigated in primary mouse microglial cells by evaluating the effect of low or high glutamate levels in NLRP3 activation [14]. In the first condition, at physiological glutamate concentrations, A_2A_ stimulation is able to reduce NLRP3 inflammasome activation and as a consequence IL-1β and IL-18 secretion, thus decreasing neuroinflammation, while in the second one, at millimolar glutamate levels, A_2A_ receptor stimulation plays an opposite proinflammatory role, suggesting that glutamate level exerts a crucial role in neuroinflammation and that the A_2A_ effect is context-dependent. These data are in agreement with a previous study in which A_2A_ receptor activation induced anti- or proinflammatory effects depending on glutamate concentration in primary microglial cells and also in animal studies evaluating traumatic brain injury (TBI) [95]. Indeed, following TBI, local glutamate levels in the brain determine whether A_2A_ receptor agonists or antagonists have a neuroprotective effect. These findings shed light on the complexities of A_2A_ receptor–glutamate connections, and suggest a novel approach for managing inflammatory pathways and minimizing brain damage by modulating A_2A_ receptor activity in response to glutamate levels in the local environment [95].

## 6. Conclusions

This review describes the effects of A_2A_ receptor activation in the context of neuroinflammation mediated by NLRP3 inflammasome and its potential to treat AD. However, there are some obstacles to overcome before a clinical application may be successful. First, it is necessary to elucidate the exact role of A_2A_ receptor blockade/stimulation in terms of time and location, considering the beneficial/harmful effects of chronic-versus-acute as well as central-versus-peripheral activation. Indeed, the expression of A_2A_ receptors changes depending on injury events and their effect may be opposite in different pathological conditions, body regions, and cell types. Second, it is necessary to carry out more studies concerning the link between the A_2A_ receptor and NLRP3 inflammasome in order to elucidate the scale of the adenosine signal in vivo. Is A_2A_ receptor activation required to boost inflammasome recruitment in order to provide tissue injury protection, or is its blocking preferable to lower NLRP3 activity and therefore organ damage? Despite the aforementioned limitations, the A_2A_ receptor and the NLRP3 inflammasome are attractive therapeutic targets to treat ailments, including neurodegenerative diseases, and the extensive work in research treatments utilizing this strategy, particularly for AD, look promising. As a result, we propose that these limitations should be addressed in order to use A_2A_ receptor antagonists as therapeutic medications.

## Data Availability

Not applicable.
